# Nanopolymers improve delivery of exon skipping oligonucleotides and concomitant dystrophin expression in skeletal muscle of *mdx *mice

**DOI:** 10.1186/1472-6750-8-35

**Published:** 2008-04-02

**Authors:** Jason H Williams, Rebecca C Schray, Shashank R Sirsi, Gordon J Lutz

**Affiliations:** 1 Drexel University College of Medicine, Department of Pharmacology and Physiology, Philadelphia, Pennsylvania 19102, USA; 2 Drexel University, Department of Biomedical Engineering, Philadelphia, Pennsylvania 19104, USA

## Abstract

**Background:**

Exon skipping oligonucleotides (ESOs) of 2'O-Methyl (2'OMe) and morpholino chemistry have been shown to restore dystrophin expression in muscle fibers from the *mdx *mouse, and are currently being tested in phase I clinical trials for Duchenne Muscular Dystrophy (DMD). However, ESOs remain limited in their effectiveness because of an inadequate delivery profile. Synthetic cationic copolymers of poly(ethylene imine) (PEI) and poly(ethylene glycol) (PEG) are regarded as effective agents for enhanced delivery of nucleic acids in various applications.

**Results:**

We examined whether PEG-PEI copolymers can facilitate ESO-mediated dystrophin expression after intramuscular injections into tibialis anterior (TA) muscles of *mdx *mice. We utilized a set of PEG-PEI copolymers containing 2 kDa PEI and either 550 Da or 5 kDa PEG, both of which bind 2'OMe ESOs with high affinity and form stable nanoparticulates with a relatively low surface charge. Three weekly intramuscular injections of 5 μg of ESO complexed with PEI2K-PEG550 copolymers resulted in about 500 dystrophin-positive fibers and about 12% of normal levels of dystrophin expression at 3 weeks after the initial injection, which is significantly greater than for injections of ESO alone, which are known to be almost completely ineffective. In an effort to enhance biocompatibility and cellular uptake, the PEI2K-PEG550 and PEI2K-PEG5K copolymers were functionalized by covalent conjugation with nanogold (NG) or adsorbtion of colloidal gold (CG), respectively. Surprisingly, using the same injection and dosing regimen, we found no significant difference in dystrophin expression by Western blot between the NG-PEI2K-PEG550, CG-PEI2K-PEG5K, and non-functionalized PEI2K-PEG550 copolymers. Dose-response experiments using the CG-PEI2K-PEG5K copolymer with total ESO ranging from 3–60 μg yielded a maximum of about 15% dystrophin expression. Further improvements in dystrophin expression up to 20% of normal levels were found at 6 weeks after 10 twice-weekly injections of the NG-PEI2K-PEG550 copolymer complexed with 5 μg of ESO per injection. This injection and dosing regimen showed over 1000 dystrophin-positive fibers. H&E staining of all treated muscle groups revealed no overt signs of cytotoxicity.

**Conclusion:**

We conclude that PEGylated PEI2K copolymers are efficient carriers for local delivery of 2'OMe ESOs and warrant further development as potential therapeutics for treatment of DMD.

## Background

Steric block oligomers such as of 2'O-methyl (2'OMe) and phosphorodiamidate morpholino (PMO) oligonucleotides possess high affinity for their complementary pre-mRNA targets and can modulate alternative splicing, correct aberrant splicing, and induce skipping or inclusion of specific exons (for reviews see [1,2]). These splice modulating oligonucleotides (SMOs) represent a powerful class of compounds with broad utility for basic and translational research and are poised to show rapid growth as pharmaceuticals. However, for many *in vivo *applications, SMOs administered alone show an inadequate delivery profile for reaching target cell nuclei, necessitating the use of carriers. Indeed, inefficient delivery of SMOs remains the foremost limitation to their usefulness as pharmaceuticals.

Duchenne muscular dystrophy (DMD) is a fatal x-linked disease caused by mutations in the gene encoding the 427 kDa membrane-associated cytoskeletal protein dystrophin, resulting in progressive body-wide muscle weakening and death usually in the early to mid third decade of life. DMD mutations are most often comprised of insertions and deletions that alter the dystrophin reading frame or encode premature stop codons [3]. These types of mutations result in production of truncated and non-functional dystrophin that is rapidly degraded.

Steric block exon skipping oligonucleotides (ESOs) have been shown in cultured mouse, canine, and human cells to cause skipping of targeted dystrophin exons, resulting in production of full-length dystrophin mRNA (minus only the skipped exons), and restoration of the reading frame [4-10]. The classical animal model for DMD, the *mdx *mouse, has a point mutation in dystrophin exon 23 that produces a premature stop codon. ESOs have been shown to promote skipping of exon 23 and concomitant dystrophin expression in skeletal muscles of *mdx *mice after both local and systemic delivery [8,11-17], and this technology in the context of therapy for DMD has been recently reviewed [18,19]. However, the efficiency of 2'OMe ESO delivery, and level of concomitant dystrophin expression in the *mdx *mouse remains relatively modest [11,13,16]. Despite the fact that ESOs without a carrier have progressed to phase I clinical trials, improved carriers must be developed before the ESO approach can be considered as a viable therapeutic for improving health and longevity in DMD patients.

The amine-rich cationic polymer poly(ethylene imine) (PEI) is a well-studied compound that is effective at condensing large plasmid DNA, enabling improved cellular uptake [20-25]. Although most often applied to plasmid delivery, recent studies have also documented PEI-enhanced delivery of small nucleic acid agents such as oligonucleotides and siRNA [26-33]. The positive surface charge of PEI-nucleic acid polyplexes interacts with negatively charged elements on the cell membrane, stimulating non-specific receptor-mediated endocytotic uptake [20,21,24,25,34,35]. Once internalized, the 'proton sponge effect' enabled by PEI's buffering capacity induces the rupturing of the endosomal compartment due to osmotic lysis [34,36-38]. While the intracellular dynamics of PEI-nucleotide complexes once released from the endosome are unknown, oligonucleotides must dissociate from PEI to reach the nucleus, as the nuclear envelope is likely impermeable to PEI. Using dual-fluorescence tracking we recently showed PEG-PEI copolymers enhanced SMO delivery to myonuclei of cultured *mdx *myofibers, while the copolymers were mainly excluded from entering the nuclei [39].

The functionality and biocompatibility of PEI is greatly improved by incorporation of the nonionic linear polymer poly(ethylene glycol) (PEG) into PEG-PEI copolymers [23,24]. The macromolecular properties of PEG-PEI-oligonucleotide polyplexes are greatly influenced by the molecular weight of PEI and nature of PEGylation, which in-turn effects transfection capacity [22,23,40]. We recently showed that PEG-PEI copolymers made of low MW PEI2K significantly improved delivery of ESOs to myofibers of *mdx *mice after intramuscular injections compared to high MW PEI25K copolymers [13]. We attributed the superior efficacy of the PEI2K-based copolymers to the low surface charge and high stability of the nanoparticles formed during complexation with ESOs [41]. Although intramuscular injection of these PEG-PEI-ESO polyplexes produced significantly more dystrophin-positive fibers than ESO alone, the level of dystrophin expression by Western blot reached only 2–5% of the normal level in TA muscles. Therefore, in this report we evaluated whether new injection regimens and further functionalization of PEG-PEI copolymers with gold nanoparticles might improve dystrophin expression. Our results show that repeat injections of small amounts of PEG-PEI-ESO are very effective at transducing dystrophin expression, reaching up to 20% of normal levels under the most favorable formulation and injection regimen.

## Results

### Induction of dystrophin expression following intramuscular injection of PEG-PEI-ESOs

**Figure 1:**
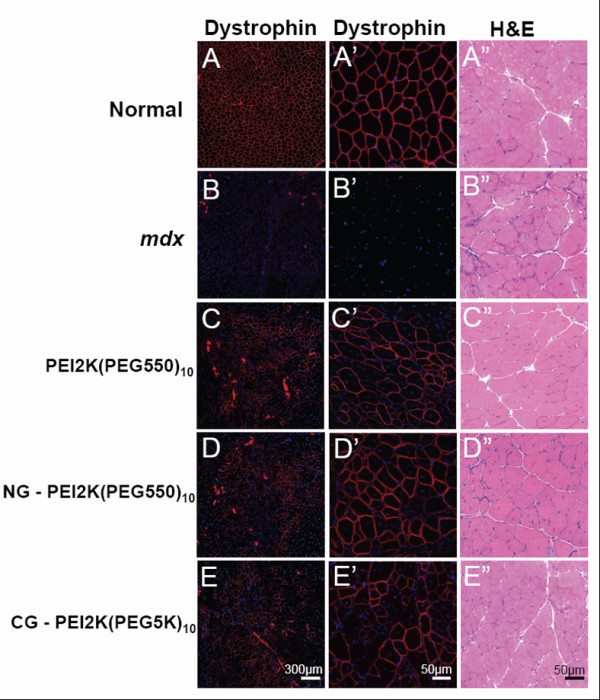
**Dystrophin induction in limb musculature of *mdx *mice after intramuscular injections of 2'OMe ESO complexed with cationic nanopolymers**. The TA muscles of *mdx *mice were given 3 weekly intramuscular injections of various PEG-PEI copolymers complexed with 5 μg of ESO and harvested 3 wks after the first injection. Dystrophin immunolabeling (Hoechst dye counterstained) at 2 different magnifications and H&E staining of serial transverse sections from TA muscles from the following groups: **(A-A") **normal age-matched controls, **(B-B") ***mdx *untreated, **(C-C") ***mdx *injected with PEI2K(PEG550)_10_-ESO, **(D-D") ***mdx *injected with NG-PEI2K(PEG550)_10_-ESO, and **(E-E") ***mdx *injected with CG-PEI2K(PEG5K)_10_-ESO.

**Figure 2:**
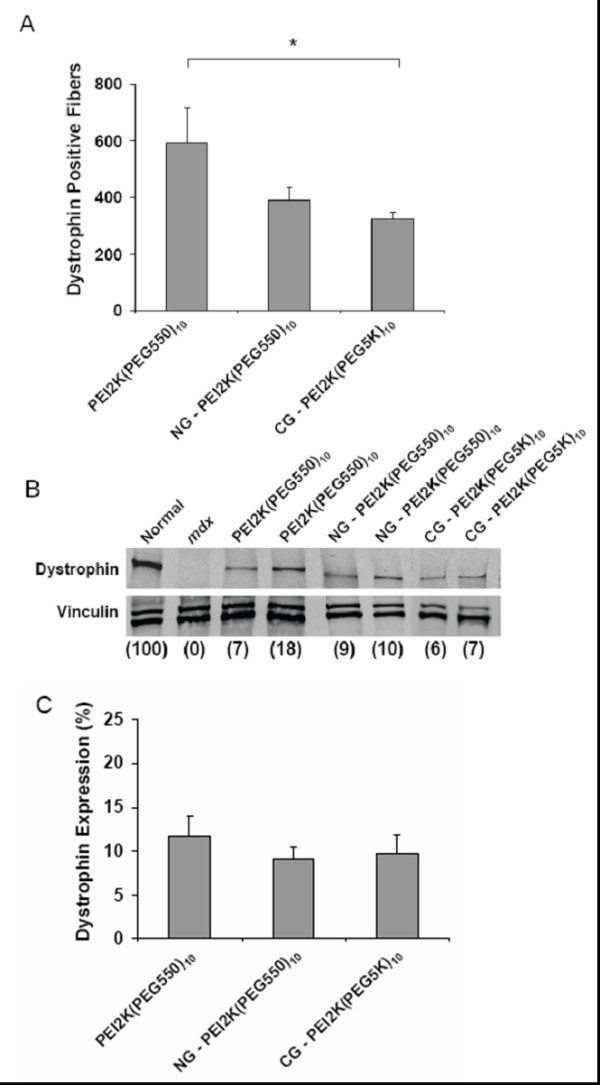
**Quantitative analysis of dystrophin expression in TA muscles of *mdx *mice after intramuscular injections of PEG-PEI-ESO polyplexes**. Muscles were analyzed following 3 weekly intramuscular injections of 5 μg of ESO complexed with either PEI2K(PEG550)_10_, NG-PEI2K(PEG550)_10_, or CG-PEI2K(PEG5K)_10 _copolymers, and harvested 3 wks after the first injection. **(A) **Number of dystrophin-positive fibers for each treatment group was obtained from whole transverse sections that were immunolabeled for dystrophin. The number of dystrophin-positive fibers was significantly lower in muscles injected with NG-PEI2K(PEG550)_10_-ESO and CG-PEI2K(PEG5K)_10_-ESO polyplexes compared with the basic PEI2K(PEG550)_10_-ESO formulation (P < 0.05, N = 4 muscles per group). **(B) **Western blots show dystrophin expression in thick (60 μm) transverse cryosections taken directly adjacent to segments used for fiber counts in panel A. Images show blots of dystrophin (top) and vinculin (bottom) obtained from the same gel. All samples contained 25 μg of total protein, and dystrophin expression as a percent of normal is indicated in parentheses below each lane. **(C) **Quantitative western analysis of dystrophin expression as a percent of the level in age-matched normal mice for each of the three PEG-PEI-ESO polyplex formulations. No significant differences were observed between treatment groups (P > 0.05; N = 4 muscles per group).

The goal of this study was to determine an effective PEG-PEI-ESO polyplex formulation and injection scheme for enhanced dystrophin expression after intramuscular injection into the TA muscle of 6–8 wk old male *mdx *mice. The ESO for all experiments was a 2'OMe oligonucleotide that was previously shown to produce specific skipping of mouse dystrophin exon 23, thereby removing a point mutation that encodes a premature stop codon [11,42]. Mice were given three weekly intramuscular injections of 5 μg of ESO complexed with either PEI2K(PEG550)_10_, NG-PEI2K(PEG550)_10_, or CG-PEI2K(PEG5K)_10 _copolymers and were analyzed for dystrophin expression at one week after the third injection. Immunohistochemistry of transverse sections showed that all three copolymer formulations produced substantially greater expression of dystrophin-positive fibers compared to muscles from un-injected *mdx *mice (Figure 1). H&E staining showed morphological integrity to be well preserved, with no overt signs of muscle necrosis or cytotoxic damage (Figure 1). Quantitative evaluation of whole muscle cross-sections showed that injections with the PEI2K(PEG550)_10_-ESO formulation produced 594 ± 120 dystrophin-positive fibers (Figure 2), which corresponds to roughly 30% of the approximately 2000 fibers in the TA muscle [11,43].

In accordance with previous studies showing improved biocompatibility and enhanced cellular uptake of gold-conjugated nanoparticles [44,45], we hypothesized that conjugation or adsorption of nanogold (NG) or colloidal (CG) to PEG-PEI copolymers would improve the potency of these carriers. Surprisingly, injections with NG-PEI2K(PEG550)_10_-ESO and CG-PEI2K(PEG5K)_10_-ESO resulted in only 380 ± 36 and 322 ± 24 dystrophin-positive fibers, respectively, both of which were significantly less than found with the unconjugated PEI2K(PEG550)_10 _copolymer (Figure 2A; P < 0.05).

To quantify dystrophin expression, Western blots were performed on whole muscle transverse sections directly serial to those on which fiber counts were obtained. Dystrophin expression following three weekly injections of the PEI2K(PEG550)_10_-ESO polyplex (5 μg ESO per injection) reached 11.2 +/- 2.4% of the level found in normal muscles (Figure 2B–C). As expected, dystrophin expression in control *mdx *muscles was not detectable, and was likely less than 1% of normal. The NG-PEI2K(PEG550)_10_-ESO and CG-PEI2K(PEG5K)_10_-ESO formulations resulted in 7.6 ± 1.0% and 9.8 ± 2.1% of the normal level of dystrophin expression, neither of which was statistically different than the unconjugated copolymer (Figure 2B–C). Although we did not perform injections of ESO without polymers in this study, we have shown in other studies that injections of 2'OMe ESO alone, under very similar dosing and injection regimens as used herein, produced very low numbers of dystrophin-positive fibers, and showed no detectable dystrophin on Western blots ([13] and Sirsi et al., manuscript submitted). Similarly, a previous study using this ESO delivered without a carrier showed no improvement in the number of dystrophin positive fibers over *mdx *control muscles [43]. The ineptitude of 2'OMe ESO delivery without a carrier in the *mdx *mouse has led to omitting ESO alone in other studies examining dystrophin induction following local and systemic delivery [11,16].

### Dose-response profile of PEG-PEI-ESO polyplexes

**Figure 3:**
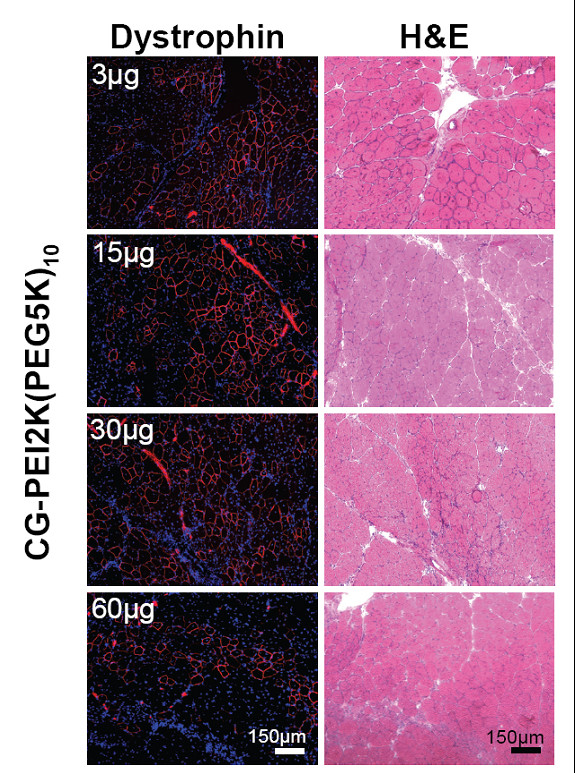
**Dose-response profile of dystrophin expression after intramuscular injections of CG-PEI2K(PEG5K)_10_-ESO polyplexes**. The TA muscles of *mdx *mice were given 3 weekly intramuscular injections of CG-PEI2K(PEG5K)_10 _copolymers complexed with 1, 5, 10, or 20 μg of ESO per injection (3, 15, 30, and 60 μg total) and harvested 3 wks after the first injection. Images show dystrophin immunolabeling (Hoechst dye counterstained) and H&E staining of serial transverse sections from TA muscles for each of the 4 polyplex doses.

**Figure 4:**
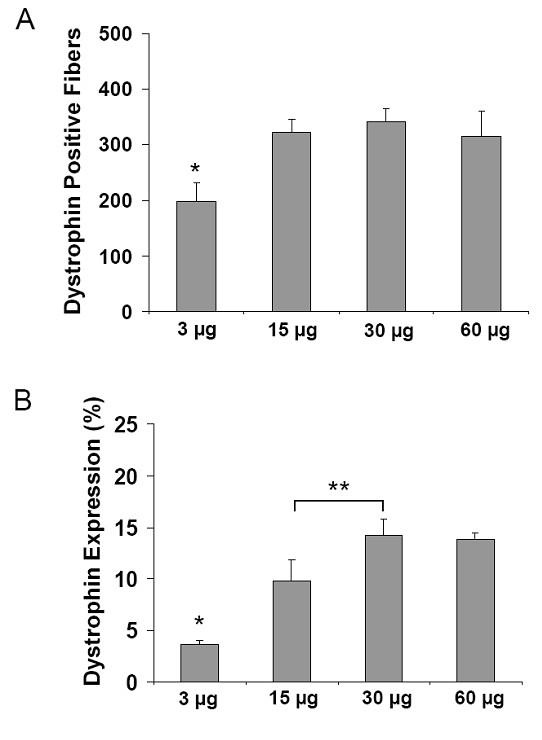
**Quantitative analysis of dose-response profile of dystrophin expression after intramuscular injections of CG-PEI2K(PEG5K)_10_-ESO polyplexes**. Muscles were analyzed following 3 weekly intramuscular injections of 1, 5, 10, or 20 μg of ESO (3, 15, 30, and 60 μg total) complexed with CG-PEI2K(PEG5K)_10 _copolymers, and harvested 3 wks after the first injection. **(A) **Number of dystrophin-positive fibers for each dosage was obtained from whole transverse sections that were immunolabeled for dystrophin. Number of dystrophin-positive fibers was significantly lower for the 1 μg injections than for all other doses (P < 0.05, N = 4 muscles per group). **(B) **Quantification of dystrophin expression as a percentage of age-matched normal mice for each of the 4 polyplex dosages based on densitometry of western blots (not shown). Samples were prepared from thick (60 μm) transverse cryosections taken adjacent to segments used for the fiber counts. Each increase in dosage resulted in significantly greater level of dystrophin expression, except between the 30 μg and 60 μg dosages which reached a plateau.

Using the same triple-injection regimen as above, the dose-response properties of CG-PEI2K(PEG5K)_10_-ESO polyplexes was evaluated over a range from 3–60 μg of total ESO. Dystrophin-positive fibers were detected at all dosages (Figure 3), but muscles injected with the smallest test dosage of ESO (1 μg × 3 injections) expressed significantly fewer dystrophin-positive fibers than doses of 15, 30, or 60 μg (Figure 4A; P < 0.05). Western analysis showed that the highest level of dystrophin induction was achieved using 5 μg of ESO per injection, resulting in 14.2 ± 1.6% expression (Figure 4B–C), with apparent saturation at higher doses.

### Longer term repeat ESO injections increase dystrophin expression

**Figure 5:**
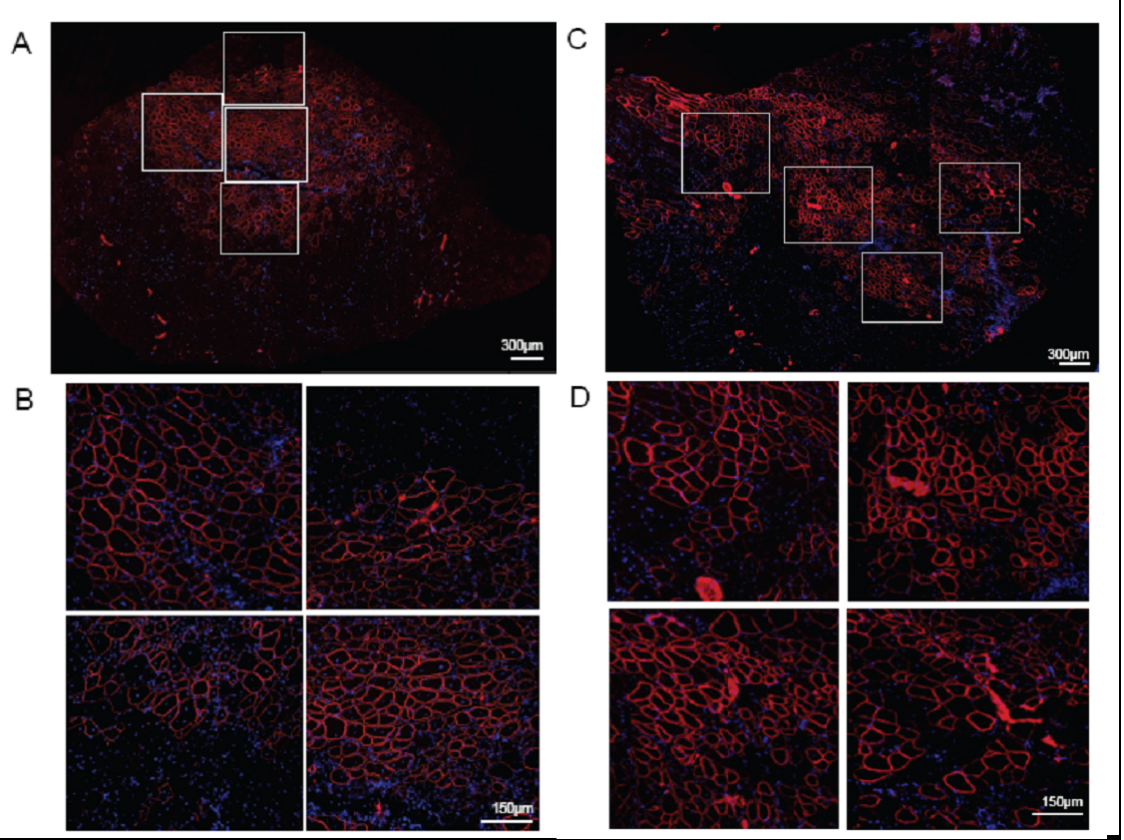
**A 6 week, repeat intramuscular injection, regimen of PEG-PEI-ESO polyplexes produced widespread manifestation of dystrophin-positive fibers**. Dystrophin immunolabeling of whole transverse sections of TA muscles of *mdx *mice is shown after 10 twice-weekly intramuscular injections of either 1 μg (**A-B**) or 5 μg (**C-D**) of ESO complexed with the NG-PEI2K(PEG550)_10 _copolymer. Muscles were harvested at 6 wks after the first of the 10 injections. High magnification views of four individual regions of each muscle section (boxed) are shown.

**Figure 6:**
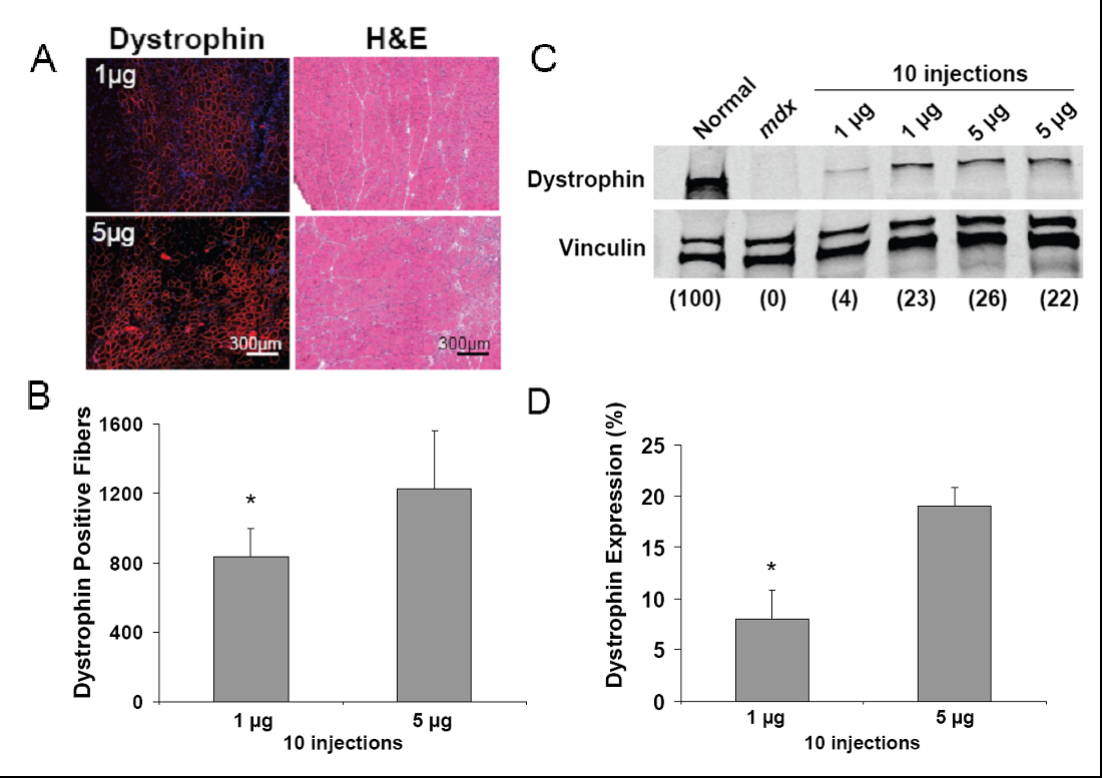
**Quantitative analysis of dystrophin expression following a 6 week, repeat intramuscular injection regimen of NG-PEI2K(PEG550)_10 _polyplexes**. Tissues were analyzed following 10 twice-weekly intramuscular injections of either 1 or 5 μg of ESO complexed with NG-PEI2K(PEG550)_10 _copolymers, and harvested 6 wks after the first injection. **(A) **Dystrophin immunolabeling (Hoechst dye counterstained) and H&E staining of serial transverse sections from TA muscles from the 10 μg (1 μg × 10 injections) and 50 μg (5 μg × 10 injections) groups. **(B) **Number of dystrophin-positive fibers, obtained from whole transverse sections immunolabeled for dystrophin, was significantly lower in muscles injected with 10 μg ESO compared with the 50 μg group (P < 0.001, N = 4 muscles per group). **(C) **Western blots showing dystrophin expression in thick (60 μm) transverse cryosections taken adjacent to segments used for the fiber counts in panel B. Images show blots of dystrophin (top) and vinculin (bottom) obtained from the same gel. All samples contained 25 μg of total protein, and dystrophin expression as a percent of normal is indicated in parentheses below each lane. **(D) **Dystrophin expression determined from Western blots reached 20% of normal levels in muscles injected with NG-PEI2K(PEG550)_10_-ESO containing 50 μg ESO, which was significantly greater than the 10 μg group (P < 0.001, N = 4 muscles per group).

In an attempt to further improve dystrophin expression, we carried out longer term repeat injection experiments using the NG-PEI2K(PEG550)_10_-ESO polyplexes. Mice were given 10 consecutive intramuscular injections of NG-PEI2K(PEG550)_10_-ESO polyplexes (1 or 5 μg of ESO per injection), with 4 days between injections, and were harvested at 6 weeks after the first injection. Immunohistochemistry of whole transverse sections showed that this protocol resulted in marked improvement in ESO delivery to myofibers, producing extensive regions of intensely labeled dystrophin-positive fibers (Figure 5). On average, muscles injected with the 1 and 5 μg of ESO per injection (10 and 50 μg total ESO over 6 weeks) contained 832 ± 167 and 1225 ± 343 dystrophin-positive fibers, respectively (Figure 6B). The number of dystrophin-positive fibers after delivery of 10 μg total ESO for 6 weeks was 2.2-fold greater than found after 15 μg of ESO for 3 weeks using the same NG-PEI2K(PEG550)_10 _copolymer. H&E staining confirmed the increased dystrophin expression following repeated injections was not associated with any overt signs of cytotoxicity (Figure 6A).

Western blots further established the potency of the 10-repeat injection regimen using the NG-PEI2K(PEG550)_10_-ESO polyplexes (Figure 6C–D). On average, muscles injected with 50 μg of ESO expressed 19.1 ± 1.8% of the normal amount of dystrophin, a level which was previously shown to be sufficient for improvement in *mdx *muscle mechanical properties [43,46,47]. The dystrophin expression after 50 μg of ESO was significantly greater than the 8.1 ± 2.8% produced with 10 μg of ESO (P < 0.05). However, there was exceptionally high variability in dystrophin expression within the 10 μg group as the lowest and highest values ranged from 4 to 23%. The peak level of 23% dystrophin expression after only 10 μg ESO injected over 6 weeks demonstrates the effectiveness of the nanopolymer-ESO formulation coupled with a low dose-high frequency delivery schedule.

### Expression of nNOS in mdx muscles treated with PEG-PEI-ESOs

**Figure 7:**
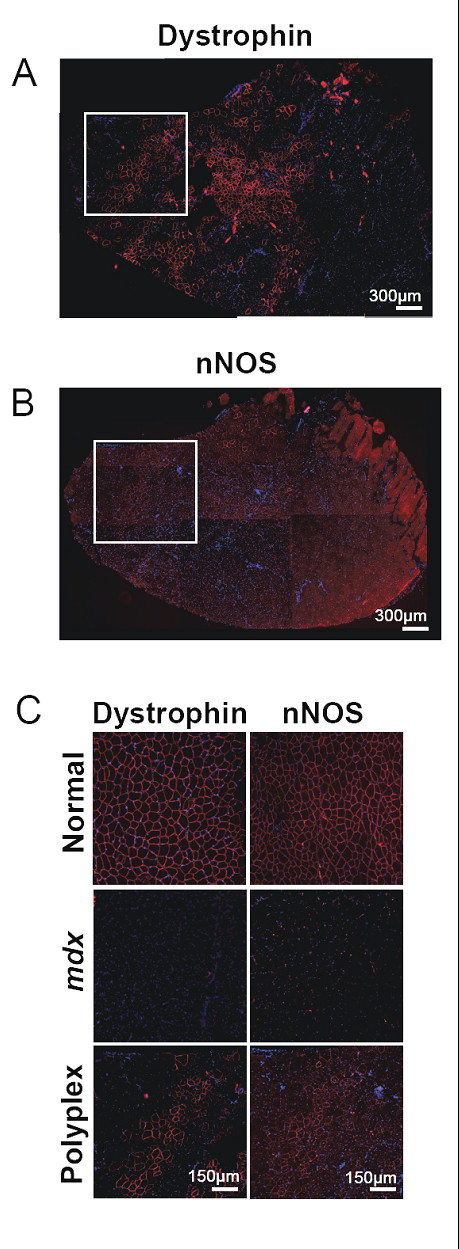
**nNOS expression in *mdx *mice after treatment with PEG-PEI-ESO**. Membrane-associated nNOS expression is upregulated in PEG-PEI-ESO treated *mdx *muscles in regions highly positive for dystrophin expression. TA muscles were given 10 twice-weekly intramuscular injections of 5 μg of ESO complexed with the NG-PEI2K(PEG550)_10 _copolymer, and harvested 6 wks after the first injection. (**A-B**) Images of serial whole transverse sections immunolabeled for dystrophin (A) and nNOS (B) show the correlation in ESO-mediated upregulation of these two membrane-associated proteins. **(C) **Higher magnification images of specific regions from panels A and B show clearly the concomitant (co-localized) upregulation of both dystrophin and nNOS in the same set of fibers of the polyplex-injected muscles. As expected nNOS expression in untreated *mdx *controls was negligible.

Muscles from polyplex-treated *mdx *mice were analyzed for nNOS expression as evidence for expression of functional dystrophin with an intact N-terminal binding domain. It was previously shown that nNOS expression is absent from muscles of *mdx *mice [48] and may be upregulated following exon-skipping restoration of dystrophin expression [11]. Therefore, we asked whether the current strategy for restoring dystrophin expression increased the level of nNOS protein localized at the myofiber membrane, where it serves to maintain normal cellular function in close association with dystrophin. Serial sections immunolabeled for dystrophin and nNOS showed a very tight correlation between dystrophin- and nNOS-positive fibers (Figure 7). Specifically, membrane-associated nNOS appeared to be expressed only in the *mdx *muscle cells that also showed enhanced dystrophin expression, indicating that ESO-expressed dystrophin was functionally intact. As expected, nNOS expression was absent from *mdx *un-injected control muscles.

## Discussion

The growing opinion that ESOs are on the path to becoming a viable therapeutic option for DMD is well supported by cell culture and animal data showing specific skipping of various targeted exons and resultant induction of nearly full-length dystrophin [4,6,7,9,11-16,49,50]. However, there is vigorous debate as to which ESO chemistry may work best, and what type of carrier compound will provide adequate delivery to body musculature. ESOs of phosphorothioate 2'OMe and PMO chemistry have been the best studied in animal models for DMD and both are currently being tested in Phase I clinical safety trials [51]. Both 2'OMe and PMO ESOs function by sterically blocking pre-mRNA target sequences, and it is thought that they may be used interchangeably once an optimal sequence (for a single chemistry) has been empirically determined [52]. However, fundamental differences in 2'OMe and PMO backbone chemistry preclude the use of a universal carrier for efficient delivery. Specifically, PMOs are synthetic compounds that are extraordinarily resistant to chemical degradation, but they are also charge neutral which limits cell surface interactions and cellular uptake. The non-degradable nature of PMOs also raises concerns over their safety after extended applications In contrast, 2'OMe ESOs are anionic RNA, and despite improved stability due to their phosphorothioate backbone, remain somewhat susceptible to degradation, while the negative charge hinders biodistribution and cellular uptake.

In this study we showed that PEG-PEI copolymers formulated with low MW PEI2K function as effective carriers for delivery of 2'OMe ESOs to myofibers of *mdx *mice after intramuscular injections, resulting in improved levels of dystrophin expression. Specifically, three weekly intramuscular injections of only 5 μg of ESO complexed with the PEI2K(PEG550)_10 _copolymer resulted in about 600 dystrophin-positive fibers and about 11% of the normal level of dystrophin expression at 3 weeks after the initial injection. Still higher levels of dystrophin expression were achieved using a twice-weekly injection regimen extended out to 6 weeks. Specifically, 10 consecutive injections of the NG-PEI2K(PEG550)_10 _copolymer complexed with 5 μg of ESO produced over 1200 dystrophin positive fibers and 20% of normal levels of dystrophin expression. In regions with the most highly transfected fibers, we observed a concomitant increase in membrane-associated nNOS, specifically in dystrophin-positive fibers, in agreement with previous reports using this ESO [11]. Our lab, as well as other, have demonstrated that intramuscular injections of 2'OMe ESO alone, using very similar conditions as used herein, produced very few dystrophin-positive fibers and negligible levels of dystrophin on western blots [13,43]. In addition, we previously showed that single injections of the PEI2K(PEG550)_10 _copolymer complexed with 20 μg of ESO produced about 460 dystrophin-positive fibers at 3 weeks after the injection, but western blots showed dystrophin expression was only 2–5% of normal levels [13]. Taken together, the current results suggest that PEG-PEI copolymers enhance dystrophin expression and that repeat injections are more effective at transfecting a greater number of muscle fibers than individual injections containing about the same amount of ESO.

Although the dystrophin expression levels shown in this report demonstrate the utility of PEG-PEI nanopolymers for delivery of ESOs, these compounds appear to be somewhat less effective than PMOs. Specifically, Alter et al [14] recently showed that single intramuscular injections of 10 μg of PMO resulted in up to 60% of the normal level of dystrophin expression, although this report appeared to provide only an estimate of efficacy and lacked statistical validation. Other recent studies of PMOs with conjugated peptide cell-targeting moieties showed impressive numbers of dystrophin-positive fibers, but did not provide a thorough evaluation of dystrophin expression by western blots [12].

The PEI2K(PEG550)_10 _and PEI2K(PEG5K)_10 _copolymers utilized in this study were previously shown to form exceptionally stable complexes when mixed with negatively charged ESO and the surface charge of the resultant nanoparticulates was relatively low [41]. We propose that the high stability and low surface charge of these polyplexes are two salient features that make them better suited for *in vivo *delivery of ESOs than high MW PEI25K-based copolymers. Specifically, the low polyplex surface charge favors biodistribution and reduces cytotoxicity, while the high stability allows the polyplex to remain associated during extracellular to intracellular trafficking.

Gold nanoparticles such as NG and CG have been shown to improve biocompatibility and enhance cellular uptake of various types of cargo in drug delivery applications [44,45,53,54]. In particular, NG conjugated to low MW PEI2K showed at least an order of magnitude greater efficiency than PEI25K and was 12 times more potent than unmodified PEI2K for delivery of plasmid DNA in cell culture [44]. Unexpectedly, our results showed that neither covalent conjugation of NG or electrostatic surface coating with CG of the PEG-PEI copolymers improved ESO delivery. Polyplex stability assays in PBS showed CG and NG caused only moderate weakening of polyplex stability (data not shown), making this an unlikely explanation for the lack of improved delivery. A possible explanation for the lack of improvement is that the CG coating was not stable enough to adhere to the copolymer during delivery. On the other hand, NG was covalently conjugated to PEG-PEI, and we postulate that its ineffectiveness was more likely due to the 1:10 NG to PEI2K ratio, which may have been too low to improve functionality.

The dystrophin expression achieved in the present study was accomplished with PEG-PEI carriers that did not appear to elicit any overt signs of cytotoxicity. This is in contrast to previous studies using non-PEGylated PEI25K as a carrier of 2'OMe ESOs, which was ineffective and resulted in significant damage following only a very limited number of injections [55,56]. Based on these results, it was concluded that cationic polymers are unsuitable for *in vivo *delivery of AO in skeletal muscle [11,57]. However, the PEI-nucleotide particles used in these previous studies undoubtedly had very high positive surface charges, because they did not contain PEG, which is known to provide steric shielding of the PEI surface charge. In addition, dispersion of the highly-charged particles after intramuscular injection is probably severely hindered by charge interactions between the PEI and negatively-charged elements within the extracellular environment. Therefore, it is not surprising that these previous muscle transfection studies with non-PEGylated PEI produced unsatisfactory results. We suggest that the combination of a low MW PEI and extensive PEGylation used presently provided a favorable formulation which was both effective and non-toxic. However, the lack of cytotoxicity observed does not preclude the possibility that some damage to muscle occurs immediately after injection, resulting in some level of degeneration-regeneration. This process may underlie to some extent the high number of dystrophin-positive fibers observed in our 6 week (10 injection) trials. Although not systematically evaluated, we have observed that short-term mechanical damage occurs in *mdx *muscles after intramuscular injections of various solutions (even saline) that does not occur in normal muscle. Because of the lack of dystrophin, *mdx *muscles are more susceptible to mechanical damage than normal muscle. This effect may be exacerbated to some extent by cationic particles, or for that matter, any type of carrier compound.

We suggest that the major limitation of the carrier-ESO formulations described in this report was inadequate carrier functionality, and not a lack of intrinsic potency of the ESO. The ESO used in the present study (designated in the literature as M23D(+02–18)) has been shown *in vitro *to predominantly produce skipping of exon 23, although some exon 22–23 double skipping does occur [42]. Moreover, in this study we showed that under the most effective condition, about 50% of fibers were dystrophin-positive, resulting in about 20% of normal dystrophin expression. This indicates that on average dystrophin-positive fibers contained about 40% of the normal level of dystrophin. A similar calculation based on data reported by Lu et al [11], and our recent study with TAT-conjugated copolymers (Sirsi et al., manuscript submitted) suggests that dystrophin per transfected fiber may reach 75% of normal levels. Thus, the main limitation with cationic carriers seems to be their poor diffusional distribution, as indicated by large regions in muscles with no apparent transfection. Thus, further improvements in carrier functionality will likely be required to enable their usage in a clinical setting for DMD. Our group recently showed that conjugation of multiple HIV-TAT epitopes to PEI2K(PEG5K)_10 _copolymers greatly improved ESO delivery, using a similar dosing and intramuscular injection regimen as reported here, resulting in up to 30% dystrophin expression (Sirsi et al., manuscript submitted). Various other types of cell targeting ligands, cell penetrating peptides, or fusogenic peptides may also be conjugated to PEI to improve functionality. Importantly, this type of peptide-PEI conjugate can likely be formulated for improved systemic delivery, which will be required to achieve meaningful therapeutic benefit. PMOs have already been shown to have limited efficacy after systemic delivery. For example, intraperitoneal injections into neonatal *mdx *mice of 5 mg/kg/week with PMO-peptide produced widespread dystrophin in diaphragm muscle, with low levels of expression observed in limb muscles [12,58].

## Conclusion

In this report, we show that PEGylated amine-rich branched cationic nanopolymers comprised of low MW PEI2K are effective carriers for delivery of ESOs to myofibers of *mdx *mice after intramuscular injections. Our results indicate that high frequency, low-dosage, long-term injection regimens using these carrier-ESO compounds provide the most favorable outcome in terms of dystrophin expression. While other studies have shown improvements utilizing gold nanoparticles, we showed no enhancement when conjugated to the current PEG-PEI formulations. Despite the lack of improvement using these novel conjugations, PEGylated PEI2K copolymers remain as one of the most efficient carriers for local delivery of 2'OMe ESOs and warrant further development as potential therapeutics for treatment of DMD.

## Methods

### Animals

Male *mdx *mice (C57BL/10ScSn-Dmd^mdx^/J) and age-matched 6–9 wk old normal mice (C57BL/10SnJ) were obtained from Jackson Laboratories (Bar Harbor, ME). All animals were housed according to NIH and University guidelines (Drexel University College of Medicine, ULAR facility, Philadelphia, PA).

### Nanopolymer synthesis

The synthesis and physiochemical characterization of the PEI2K(PEG550)_10 _and PEI2K(PEG5K)_10 _copolymers were previously described [41,59]. Copolymers are designated using a nomenclature where the subscript indicates the number of PEG chains grafted per molecule of PEI. For example, PEI2K(PEG550)_10 _indicates 10 PEG chains of 550 daltons grafted to each 2 kDa PEI molecule. Nanogold (NG) particles were conjugated to PEI primary amine groups on PEI2K(PEG550)_10 _coploymers using the Sulfo-*N- *Hydroxy-Succinimido Nanogold labeling reagent (Nanoprobes, Yaphank, NY). In this reaction, 75 nmols of PEI2K(PEG550)_10 _was mixed with 6 nmols of NHS-nanogold in 780 μl of sterile water (pH = 8.0). The solution was incubated for 24 hours on ice, frozen, and subsequently freeze-dried and stored at -20°C. Adsorption of colloidal gold (CG) to PEI2K(PEG5K)_10 _was performed by mixing 300 μl of 5 nM CG particles (Sigma-Aldrich) with 30 mg of PEI2K(PEG5K)_10 _(in 1 ml DI H_2_0) and incubating at 4°C overnight. The polymer solution was subsequently freeze-dried and stored at -20°C. The NG and CG labeled copolymers are designated as NG-PEI2K(PEG550)_10 _and CG-PEI2K(PEG5K)_10_, respectively.

### Preparation of PEG-PEI-ESO polyplexes

The ESO used in all experiments was a 20-mer oligoribonucleotide (5'-GGCCAAACCUCGGCUUACCU-3') previously shown to cause skipping of dystrophin exon 23 in *mdx *mice [11,16,42]. During synthesis (Trilink, San Diego, CA) each base was phosphorothioated and contained a methoxy group at the 2' carbon. PEG-PEI-ESO polyplexes were prepared at a nitrogen to phosphate ratio of 5 (N:P = 5); where N represents moles of amine on PEI, and P represents moles of phosphate on ESO. Polyplexes were formed by the addition of the PEG-PEI copolymer solution to the ESO solution (in sterile saline). Polyplex solutions were vortexed briefly, sonicated for 30 min using a bath sonicator, incubated on ice for 30 min, and used immediately.

### Intramuscular injections of PEG-PEI-ESO polyplexes

Male *mdx *mice (6–9 weeks of age) were anesthetized with ketamine/xylazine and shaved for visualization of hindlimb muscles. A 15 μl volume of PEG-PEI-ESO polyplex solution at various concentrations was injected bi-laterally into the mid-belly portion of TA muscles using a 31 gauge insulin syringe. After recovery from anesthesia, mice were returned to normal cage activity.

We used both a 3 wk and 6 wk polyplex injection schedule as follows. For the 3 wk groups, mice were injected on days 0, 7, and 14; and were harvested on day 21. In one series of experiments muscles were injected with 5 μg of ESO (15 μg total) complexed with either the PEI2K(PEG550)_10_, NG-PEI2K(PEG550)_10_, or CG-PEI2K(PEG5K)_10 _copolymers. For dose-response analysis of a single polyplex, muscles were injected with 1, 5, 10, or 20 μg of ESO (3, 15, 30, and 60 μg total) complexed with the CG-PEI2K(PEG5K)_10 _copolymer. For the 6 wk groups, mice were injected on days 0, 4, 8, 12, 16, 20, 24, 28, 32, and 36; and were harvested on day 42. For this experiment, muscles were injected with either 1 or 5 μg of ESO per injection (10 and 50 μg total) complexed with the NG-PEI2K(PEG550)_10 _copolymer. For all groups, 2 mice (N = 4 muscles) were analyzed.

### Muscle harvest, immunohistochemistry, histology, and fiber counts

At designated time points mice were killed and TA muscles were removed, pinned to parafilm-covered cork, snap frozen in liquid N_2_-cooled isopentane, and stored at -80°C. Control muscles were harvested from uninjected age-matched *mdx *and normal mice. Transverse frozen sections (10 μm) were cut from the belly of each TA muscle using a cryostat (Leica CM 3050 S, Bannockburn, IL) and melted onto slides for immunohistochemistry and histochemistry. Thick (60 μm) transverse sections, immediately adjacent to the thin sections were cut in the cryostat and placed in 1.5 mL centrifuge tubes on dry ice, and stored at -80°C for subsequent western analysis.

For immunolabeling, muscle sections were blocked with 10% normal goat serum in 1% BSA/PBS for 1 h and then incubated for 1 h in rabbit polyclonal anti-dystrophin (1:200; Abcam, Cambridge, MA) or anti-nNOS (1:125; Invitrogen, Carlsbad, CA). The secondary antibody was Cy3-Anti-Rabbit IgG (1:500; Jackson Immunoresearch, West Grove, PA). Slides were coverslipped with Vectashield mounting medium with DAPI (Vector Laboratories, Burlingame, CA) and imaged (4/10/20× objective; Olympus, AX70, Melville, NY). Composite images of entire transverse sections were constructed from overlapping low magnification images using Adobe Photoshop (Adobe, San Jose, CA). Fiber counts of dystrophin-positive fibers were obtained using the cell counter function of ImageJ software (rsb.info.nih.gov/ij/plugins/cell-counter.html).

### Western analysis of dystrophin expression

Sections of frozen TA muscles (60 μm) were extracted in 1.5 ml centrifuge tubes by pipetting up and down in 50 μl of protein extraction buffer containing 125 mM Tris (pH 6.8), 4% SDS, 2 M Urea, 5% 2-mercaptoethanol, 10% glycerol, 5 μl of protease inhibitor cocktail (Sigma, St. Louis, MO) and protease inhibitors calpeptin (100 nM; Calbiochem, San Diego, CA) and calpain inhibitor I (25 μM; Calbiochem, San Diego, CA). The extract was incubated on ice (15 min), boiled (5 min), and centrifuged (4000 × g for 5 min), and the supernatant was transferred to a clean tube. Protein concentration was measured in extracts using the Coomassie assay (Pierce, Rockford, IL) and an equal volume of SDS-PAGE sample buffer (125 mM Tris (pH 6.8), 4% SDS, 5% 2-mercaptoethanol, 10% glycerol and 0.05% bromophenol blue) was added to the extract. Samples containing 25 μg of total protein were loaded onto pre-cast SDS-PAGE gels (3% stacking: 7.5% resolving; Bio-Rad, Hercules, CA) and run at 150 V for 75 min. Gels were transferred to nitrocellulose at 30 V for 16 h and membranes were stained with Ponceau S (Sigma, St. Louis, MO) to visualize proper transfer and even loading. Membranes were cut to allow separate immunoblotting of dystrophin and vinculin and blocked in Odyssey blocking buffer (LI-COR Biosciences, Lincoln, NE) for 1 h. Membranes were incubated for 1 h in mouse monoclonal anti-dystrophin (MANDYS8; Sigma, St. Louis, MO) and anti-vinculin (VIN1; Sigma, St. Louis, MO) at dilutions of 1:400 and 1:2000, respectively. Donkey anti-mouse IRDye 800 CW secondary antibody (LI-COR Biosciences, Lincoln, NE) was applied for 1 h and membranes were scanned on an Odyssey Infrared Imaging System following multiple TBS-T/TBS washes. Odyssey imaging software was used for densitometry and the integrated intensity of sample bands was used for calculating the percentage of dystrophin expression as compared to the normal muscles. For each muscle, 2–3 separate 60 μm sections were extracted and used in western analysis.

### Statistical Analysis

All data are reported as mean values ± SEM. Statistical differences between treatment groups were evaluated by ANOVA (Statview; SAS Institute, Cary, NC).

## List of abbreviations

ESO, Exon skipping oligonucleotide; 2'OMe, 2'-O-Methyl; PMO, Phosphorodiamidate Morpholino Oligonucleotide; CPP, Cell Penetrating Peptide; DMD, Duchenne Muscular Dystrophy; PEI, Poly(ethylene imine); PEG, Poly(ethylene glycol); N:P, ratio of PEI nitrogen to ESO phosphate; TA, Tibialis Anterior; NG, nanogold; CG, Colloidal gold.

## Competing interests

The author(s) declare that they have no competing interests.

## Authors' contributions

JHW carried out western and immunoflourescence analysis, assisted with harvesting of muscle tissue, participated in the design of the study and drafted the manuscript. RCS maintained animal colonies, carried out injections and harvesting of tissues and participated in design of the study. SRS carried out chemical synthesis and functionalization of copolymers and contributed to the conception of the study. GJL conceived the study, drafted the final manuscript and participated in all stages of the work. All authors read and approved the final manuscript.
